# *Acanthamoeba castellanii* as a model for unveiling *Campylobacter jejuni* host–pathogen dynamics

**DOI:** 10.3389/fcimb.2025.1583830

**Published:** 2025-06-18

**Authors:** Fauzy Nasher, Burhan Lehri, Richard A. Stabler, Brendan W. Wren

**Affiliations:** Department of Infection Biology, London School of Hygiene and Tropical Medicine, London, United Kingdom

**Keywords:** *Campylobacter jejuni*, Acanthamoeba, mitochondria, DSB = double-strand break, host pathogen environment interactions, Warburg-like metabolism

## Abstract

The persistence of the major enteric pathogen *Campylobacter jejuni* in the natural environment, despite being microaerophilic, remains unsolved. Its survival in the natural atmospheric environment likely stems from several factors, including interactions with amoebae. *C. jejuni* transiently interacts with Acanthamoeba, and this is thought to provide protection against unfavorable atmospheric conditions and subsequently prime the bacteria for interactions with warm-blooded hosts. Acanthamoeba play vital roles in microbial ecosystems by preying on bacterial species, some of which are clinically important. We analyzed the whole transcriptome of *A. castellanii* infected with *C. jejuni* 11168H. Our findings provide evidence that infection of *A. castellanii* with *C. jejuni* triggers distinct and reproducible cellular responses. Upregulated genes were associated with protein synthesis, DNA damage and repair, gluconeogenic pathways, and protein folding and targeting, while downregulated genes were involved in calcium ion transport, osmotic stress response, energy reserve metabolic processes, and protein hydroxylation. From these data, we characterized Cj0979c, named here *C. jejuni* endonuclease (CjeN), which induces DNA damage in *A. castellanii*. High-resolution microscopy revealed an unexpected association between *C. jejuni* and host mitochondria, while infected cells show elevated cytosolic calcium levels and metabolic changes favoring “Warburg-like” metabolism. *A. castellanii* cells showed increased lactate production, which was subsequently depleted, suggesting that this host metabolic by-product may support *C. jejuni* survival. These findings identify an unexpected interaction between amoebae and a microaerophilic bacterium and provides a useful model for further research on host–pathogen interactions.

## Introduction

The long-term relationship between amoebae and bacteria holds significant implications for the evolution of pathogens and has been termed the eco–evo perspective of bacterial pathogenesis (Pallen and Wren, 2007; Berlanga et al., 2021). Amoebae are abundant unicellular organisms found in diverse environments that predate bacteria, influencing the evolution of both host and prey (Khan and Siddiqui, 2014). Free-living amoebae, such as Acanthamoeba, play essential roles in aquatic and terrestrial ecosystems, shaping microbial communities and nutrient cycling dynamics (Henriquez et al., 2020). Notably, Acanthamoeba exhibit two distinct life cycles: an active phagotrophic trophozoite stage and a low-metabolic, resilient cyst stage, aiding survival under adverse conditions (Siddiqui and Khan, 2012).

The co-evolution of bacteria and amoebae often precedes bacterial interactions with warm-blooded hosts (Shi et al., 2021), with amoebae evolving mechanisms to ingest and kill bacteria, while bacteria develop strategies to resist predation and, at times, kill amoebae hosts (Henriquez et al., 2020; Shi et al., 2021). This microbial dynamic mirrors the intricate interplay observed between phagocytes of the warm-blooded host’s immune system and pathogenic bacteria (Mungroo et al., 2021; Price Christopher et al., 2024).

*C. jejuni*, a microaerophile bacterium, challenges conventional understanding by persisting in the environment outside avian and mammalian hosts (Elmi et al., 2020). *C. jejuni* is the most frequent cause of bacterial gastrointestinal infection globally (Kaakoush et al., 2015). Despite being a leading cause of bacterial gastroenteritis, *C. jejuni* is rarely transmitted between humans, and its high prevalence in human infection is difficult to explain. While the consumption and handling of poultry contribute significantly to human infections (Lehri et al., 2024), the transmission routes to poultry from the environment remain unclear. This gap in our understanding of the prevalence and transmission of this major pathogen has been referred to as the “*Campylobacter* conundrum” (Jones, 2001). Amoebae like Acanthamoeba have been proposed as a natural reservoir for *Campylobacter*, providing a niche and protection against potential toxic atmospheric conditions (Axelsson-Olsson et al., 2005; Bare et al., 2010; Vieira et al., 2015). Additionally, some strains of *C. jejuni* exhibit hyper-aerotolerance, allowing them to survive in oxygen-rich environments (Oh et al., 2015) and potentially withstand higher levels of reactive oxygen species (ROS) during their intra-amoebic lifestyle. Acanthamoeba transiently host *C. jejuni*, priming the bacterium and facilitating its subsequent invasion of human epithelial cells and re-invasion of amoebae (Nasher et al., 2022; Nasher and Wren, 2022). Therefore, exploring Acanthamoeba–*C. jejuni* interactions offers a promising model for studying the adaptation, persistence, and infection dynamics of this enigmatic pathogen.

In the present study, we investigate the interaction between *A. castellanii* and *C. jejuni* to explore how this environmental host may influence bacterial adaptation and survival. Using transcriptomic profiling combined with complementary functional and imaging assays, we aim to elucidate the cellular and molecular mechanisms underpinning this interaction. These findings contribute to a broader understanding of microbial evolution, environmental persistence, and potential pathways of transmission for this globally significant pathogen.

## Results

### *A. castellanii* differentially regulates its genes in response to *C. jejuni* infection

To uncover the response of *A. castellanii* to intracellular *C. jejuni*, we conducted whole-genome transcriptome analyses of *A. castellanii* at 4 h post-infection with *C. jejuni* 11168H. There was a significant difference (*p* < 0.05) in gene expression between *A. castellanii* infected with *C. jejuni* and the control; a total of 196 genes were differentially transcribed (>2-fold; *p*-value < 0.05): 26 were up-regulated while 170 were down-regulated (**Supplementary File 1**).

**Figure 1:**
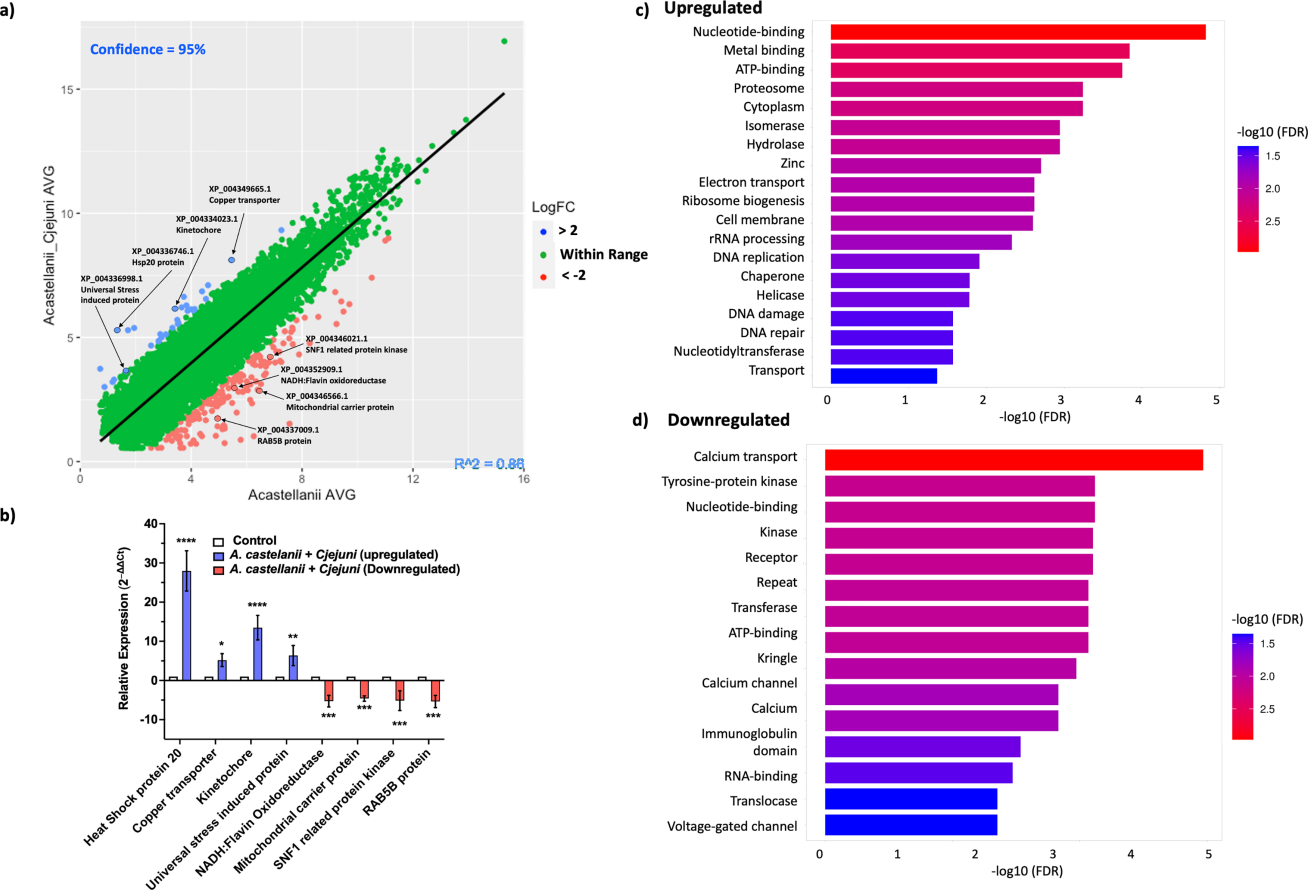
Validation and enrichment analysis of RNA-seq data. **(a)** A scatter plot displaying the average fold change between *A. castellanii* infected with *C. jejuni* versus *A. castellanii* alone 4 h post-infection. Upregulated genes are indicated in blue, while downregulated genes are shown in red. **(b)** Real-time RT-qPCR analysis of *A. castellanii* infected with *C. jejuni* 11168H. Error bars represent standard deviation (SD). **(c)** Bar plot representing the enrichment of upregulated Gene Ontology (GO) terms. **(d)** Bar plot illustrating the enrichment of downregulated GO terms. Enriched terms are indicated with colors corresponding to the significance level [−log10(FDR)]. Plots were generated using ShinyGO 0.80 (Ge et al., 2020). *p< 0.05, **p<0.002, *** p<0.0002, ****p<0.0001.

To verify our RNA-seq results, we selected a subset of genes for real-time reverse transcription polymerase chain reaction (RT-PCR) based on their relevance to stress responses, intracellular adaptation, and cellular homeostasis—key themes that emerged from our transcriptomic analysis and align with the biological context of *A. castellanii* harboring *C. jejuni*. RNA from *A. castellanii* harboring *C. jejuni* was extracted independently of the RNA-seq experiments for real-time RT-PCR. Genes that were verified are highlighted in **Figure 1a**. Upregulated genes encode heat shock protein 20 (*hsp20*) (XP_004336746.1), an adenosine triphosphate (ATP)-independent chaperone that was shown to be associated with encystation (Wang et al., 2020); a copper transport accessory protein (XP_004349665.1), possibly involved in copper homeostasis; and a kinetochore like protein (XP_004334023.1). Kinetochore-like proteins are not well-defined; however, kinetochores themselves are protein complexes that assemble on chromosome during cell division, DNA repair, and cell signaling (Cheeseman and Desai, 2008). Finally, we verified upregulation of a gene encoding for universal stress domain containing protein (USP) (XP_004336998.1). USP may provide a general “stress endurance” activity and enhance survival during prolonged exposure to stress (Luo et al., 2023).

For downregulated genes, we verified genes encoding NADH:flavin oxidoreductase (XP_004352909.1), a free flavin reductase by NADH; mitochondrial carrier protein (MCP) (XP_004346566.1); MCP transport nucleotides; amino acids; carboxylic acids; inorganic ions; and cofactors across the mitochondrial inner membrane, thereby connecting metabolic pathways of the cytoplasm with those of the mitochondrial matrix (Palmieri and Pierri, 2010). Sucrose non-fermenting 1-related protein (SNF1) (XP_004346021.1) and SNF1 kinases are widely attributed to controlling various nutrient-responsive and cellular developmental processes within the cell (Hardie, 2007), and lastly, RAB5B protein (XP_004337009.1) is a Ras-related protein that is involved in early endosome movement and is necessary for the fusion of vesicles (Pensalfini et al., 2021).

Real-time RT-qPCR analysis revealed a statistically significant (*p* < 0.05) upregulation in the expression levels of genes encoding XP_004336746.1 (∼28-fold), XP_004349665.1 (∼5-fold), XP_004334023.1 (∼12-fold), and XP_004336998.1 (∼7-fold) and the downregulation of genes encoding XP_004352909.1 (∼5-fold), XP_004346566.1 (∼4-fold), XP_004346021.1 (∼5-fold), and, finally, XP_004337009.1 (∼5-fold) (**Figure 1b**).

To further explore the functional implications of these changes, we conducted Gene Ontology (GO) enrichment analyses of both upregulated (**Figure 1c**) and downregulated (**Figure 1d**) genes. The analyses revealed several significantly enriched GO terms associated with biological processes, molecular functions, and cellular components. Among the upregulated processes, nucleotide binding, DNA replication, nucleotide excision repair, and RNA processing were observed. The downregulated genes were enriched for functions related to calcium transport, receptor activity, and ATP binding (**Supplementary File 2**).

### CjeN *(cj0979c)*, a *C. jejuni* endonuclease, induces double-stranded breakages during infection

**Figure 2:**
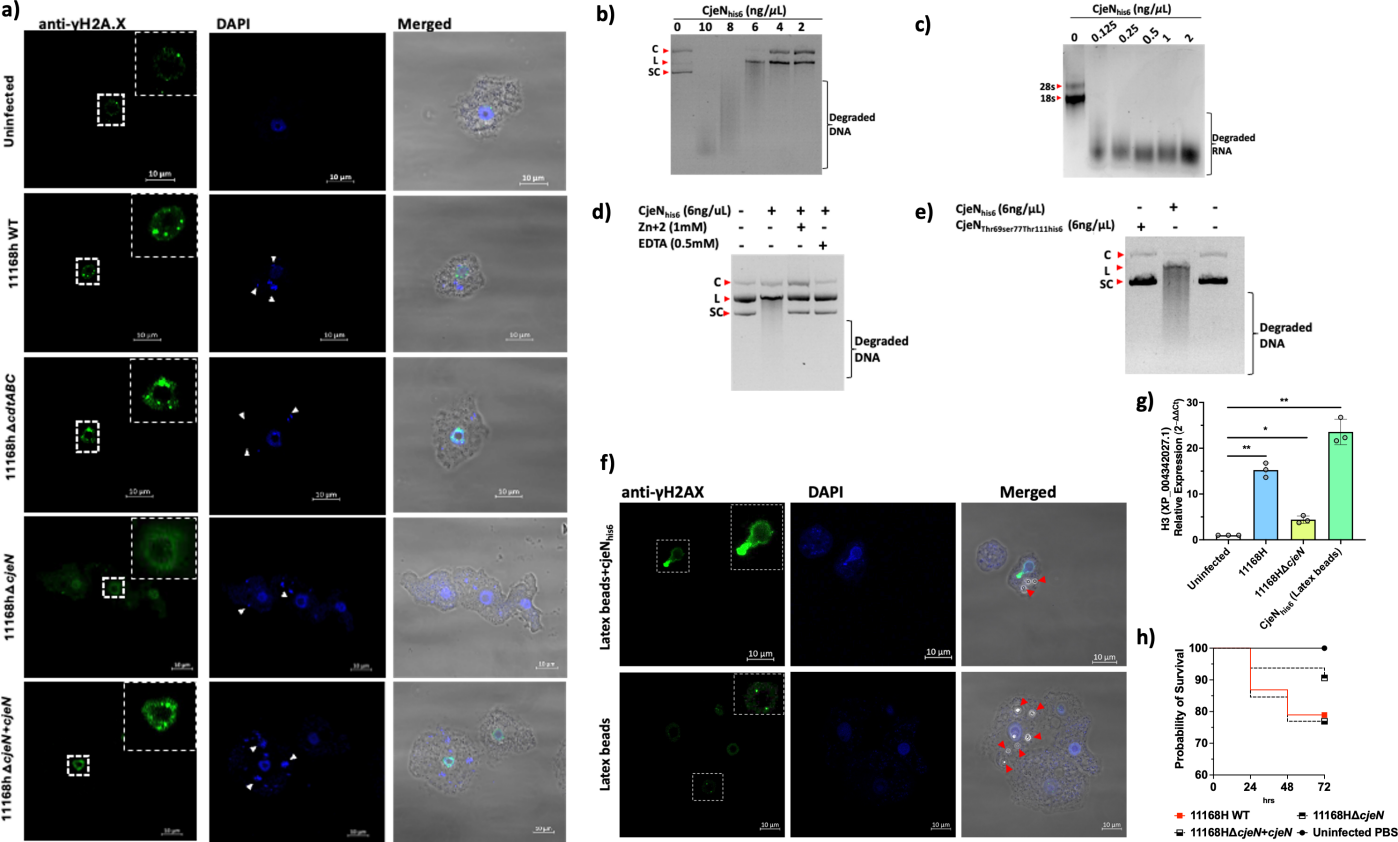
Mechanisms of DNA damage in *A. castellanii*. **(a)** Anti-γHA2.X biomarker of double-stranded breakages (DSBs) revealed that *C. jejuni* 11168H wild type, 11168HΔ*cdtABC*, and 11168HΔ*cjeN*+*cjeN* mutant cause multiple DNA damage at 4 h post-infection relative to uninfected cells and 11168HΔ*cjeN.* **(b)** CjeN nuclease activity on plasmid double-stranded DNA; smear indicates degraded DNA (C; circular, L; linear, SC; supercoiled). **(c)** CjeN activity of RNA. **(d)** Effect of zinc (Zn^+2^) or ethylenediaminetetraacetic acid (EDTA) on CjeN nuclease activity. **(e)** Effect of mutation to the active site of CjeN (CjeN_Thr69Ser77Thr111his6_) on nuclease activity. **(f)** CjeN adsorbed onto latex beads and its activity was monitored *in vivo*. **(g)** Relative expression of H3 histone family. **(h)** *G. mellonella* infected with *C. jejuni* 11168H wild type, 11168HΔ*cjeN*, or 11168HΔ*cjeN* compared to PBS alone (*n* = 120). Significance levels are indicated as follows: *p < 0.05, **p < 0.001. Error bars represent standard deviation (SD). For images: The amoebae are visible in the transmitted-light channel (labeled: Merged); nucleic acid and *C. jejuni* cells are visible in blue channel (labeled: DAPI Ex/Em 350/465 nm); anti-γHA2.X in the green channel (labeled: Anti-γHA2.X Ex/Em 493/518 nm). Arrows indicate bacteria (white) or latex beads (red); images are representation of three independent biological replicates; images were captured with ×63 oil objective Scale bar: 10 μm.

Our RNA-seq data indicated that *A. castellanii* infected with *C. jejuni* upregulated pathways that are involved in DNA damage and repair. To confirm this, we used anti-γHA2.X as a biomarker for DNA damage and monitor DNA double-stranded breaks (DSBs) (**Figure 2**).

Our findings revealed that *C. jejuni* 11168H causes multiple DSBs to *A. castellanii* DNA (**Figure 2A** **).** Similar to other Gram-negative pathogens, *C. jejuni* synthesizes a cytolethal distending toxin (CDT), which was reported to cause direct DNA damage (Lara-Tejero and Galan, 2000), thereby activating DNA damage checkpoint pathways. Comprising three protein subunits, CDT includes CdtA, CdtB, and CdtC, with CdtB serving as a nuclease. To elucidate the mechanism by which *C. jejuni* induces DSBs in *A. castellanii*, we generated a defined *cdtABC*-deficient mutant of *C. jejuni* 11168H **[** **Supplementary File 3** (**Supplementary Figure S3** **)]**. Subsequently, we infected *A. castellanii* with this 11168HΔ*cdtABC* mutant and DSBs (γHA2.X) were monitored (**Figure 2A**). Intriguingly, despite the absence of the *cdtABC* genes, multiple DSBs were still observed in *A. castellanii* infected with the *C. jejuni* 11168HΔ*cdtABC* mutant. This suggested that *C. jejuni* 11168H employed other mechanisms to induce DSBs.

Previously, RNA-seq analyses of intra-amoebic *C. jejuni* showed upregulation of an uncharacterized gene, *cj0979c*, which was predicted to be a secreted endonuclease (Nasher et al., 2022). In the current study, we sought to determine whether *cj0979c* was involved in the formation of DSBs in the DNA of *A. castellanii.* A protein BLAST search (https://blast.ncbi.nlm.nih.gov/) of *cj0979c* revealed shared identities with *Staphylococcus aureus* Nuc1 (32.80%) and *Bacillus subtilis* 168 YncB (31.30%) proteins **[** **Supplementary File 3** (**Supplementary Figure S2** **)]**. Both Nuc1 and YncB are sugar non-specific extracellular endonucleases (Rangarajan and Shankar, 2001). To explore the role of *cj0979c* (now designated as *Campylobacter jejuni* endonuclease, CjeN) in inducing DSBs, we generated *C. jejuni* 11168HΔ*cjeN*-deficient strain and infected *A. castellanii* with this mutant. Interestingly, no significant DSBs (γHA2.X) were detected in *A. castellanii* infected with this mutant strain, and this phenotype was restored when *cjeN* was complemented with a copy in the rRNA gene clusters on the chromosome (**Figure 2a**).

We sought to characterize CjeN; we therefore expressed recombinant CjeN_his6_ that lacked the first 39 amino acids [predicted signal peptide (SignalP 5.0)] (Almagro Armenteros et al., 2019). Our results confirmed that CjeN is a nuclease and was capable of digesting double-stranded DNA (plasmid DNA) and RNA non-specifically in the presence of 130 µM calcium (Ca^2+^) and 5 mM magnesium (Mg^+2^) (**Figures 2b, c**). Notably, CjeN digestion of DNA produced the linear form of the plasmid DNA, a characteristic of endonucleases. Furthermore, CjeN activity was found to be inhibited by zinc (Zn^+2^) or EDTA, a metal chelator (**Figure 2d**). Zn^+2^ is known to hinder Ca^+2^- and Mg^+2^-dependent endonuclease activity (Torriglia et al., 1997) by competing for the catalytic site. Multiple sequence analysis (MSA) of CjeN, Nuc1, and YncB identified conserved catalytic sites [CjeN; Arginine69, Glutamic acid77, and Arginine111 (**Supplementary Figure S2**)], and we generated a recombinant protein with substitution to all three sites, CjeN_Thr69ser77Thr111his6_, and confirmed that mutation to these sites affects CjeN nuclease activity (**Figure 2e**). Next, we sought to confirm CjeN endonuclease activity *in vivo* in the absence of *C. jejuni*. We immobilized recombinant CjeN_his6_ onto latex beads and incubated approximately 0.3 ng of the protein with *A. castellanii*. Subsequently, we observed DSBs indicated by γHA2.X, suggesting that CjeN was likely responsible for inducing DNA damage in *A. castellanii* host (**Figure 2f**). Additionally, through RT-qPCR analysis, we found that the relative expression of *A. castellanii* H3, histone family member (XP_004342027.1) was upregulated in the presence of both *C. jejuni* 11168H and recombinant CjeN_his6_, consistent with our RNA-seq analyses (**Figure 2g**). The *G. mellonella* infection model also showed a non-statistically significant (*p >* 0.05) ∼10% reduction in killing when infected with *C. jejuni* 11168HΔ*cjeN* relative to the wild type and 11168HΔ*cjeN* +*cjeN* complemented strain (**Figure 2h**).

### *C. jejuni* associates and alters host mitochondria dynamics

**Figure 3:**
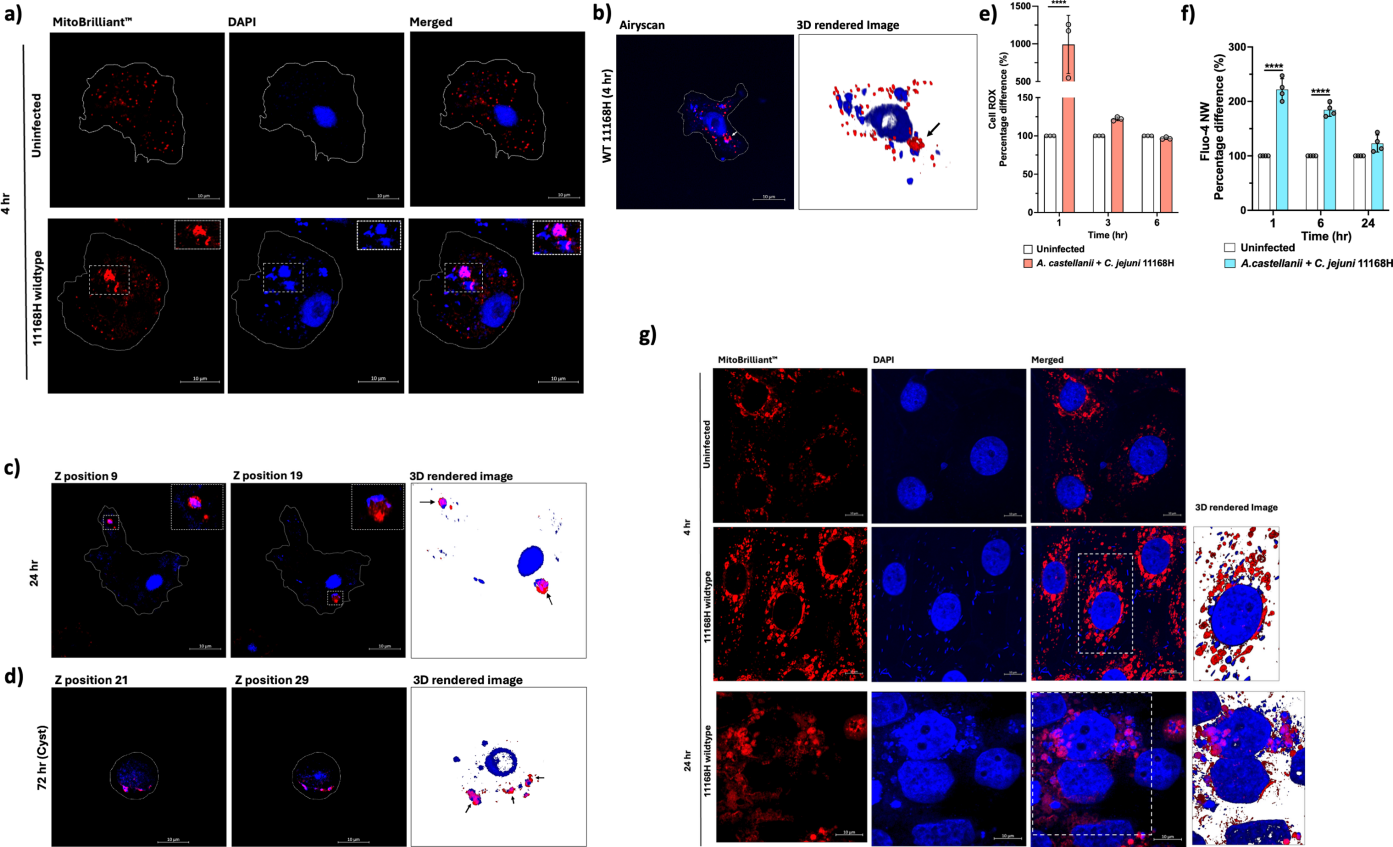
Mitochondrial alterations, ROS, and calcium dynamics in *A. castellanii* infected with *C. jejuni.* **(a)** Confocal microscopy images comparing mitochondria in uninfected *A. castellanii* (above) with those infected with *C. jejuni* for 4 h (below). **(b)** Volume view of confocal Z-stack image showing *A. castellanii* infected with *C. jejuni*, highlighting the altered mitochondrial morphology. **(c)** Z-stack positions illustrating *A. castellanii* mitochondria dynamics at 24 h post-*C. jejuni* infections. **(d)** Z-stack positions depicting *A. castellanii* cysts at 72 h post-*C. jejuni* infection. **(e)** Measurement of reactive oxygen species (ROS) using CellROX™ Deep Red, with pale pink bars representing *A. castellanii* infected with *C*. *jejuni* and white bars representing uninfected cells. **(f)** Measurement of cytosolic calcium (Ca^+2^) levels using Fluo-4 NW Calcium Assay. ROS and Ca^+2^ data are presented as percentage. **(g)** Images comparing mitochondria in uninfected human intestinal epithelial T84 cells with those infected with *C. jejuni* at 4 and 24 h (arrows indicate *C. jejuni* and mitochondria association). Mitochondria were stained with Mitobrilliant™ Red (labeled: Mitobrilliant ™ Red); nucleic acid and *C. jejuni* cells are visible in the blue channel (labeled: DAPI). Scale bar: 10 μm; *A. castellanii* were imaged at 100×, and human T84 cells were imaged at 63×. All images are representation of three biological replicates. Significance levels indicated as ****p < 0.0001. Error bars represent standard deviation (SD).

We observed that GO terms related to mitochondrial function were enriched, including GO:0005759 (mitochondrial matrix), GO:0009055 (electron carrier activity), GO:0022900 (electron transport chain), GO:0006099 (tricarboxylic acid cycle), GO:0005524 (ATP binding), and GO:0016887 (ATPase activity). Conversely, there was downregulation of GO:0000281 (mitochondrial membrane). Given this, we decided to further investigate mitochondria (**Figure 3**).

We employed Mitobrilliant™ 646 (excitation 655/emission 668), a mitochondria-specific stain that relied on mitochondria membrane potential (ΔΨm) for uptake and confocal microscopy with Airyscan to facilitate our analysis. *Acanthamoeba* spp. are recognized for possessing an exceptionally high number of mitochondria (Siddiqui and Khan, 2012), typically distributed uniformly within the cell. Remarkably, in *A. castellanii* cells infected with *C. jejuni*, we observed a distinct alteration in mitochondrial morphology, with mitochondria appearing fused (**Figure 3a**). Notably, these fused mitochondria exhibited close association with bacteria (**Figures 3a, b**). This phenomenon persisted even at 24 h post-infection (**Figure 3c**) and, strikingly, even within *A. castellanii* cysts containing *C. jejuni* at 72 h post-infection (**Figure 3d**), indicating the continuity of this phenomenon throughout the infection process. Interestingly, this phenomenon of mitochondria association was not observed with *C. jejuni* 11168HΔ*ciaB* (a putative secreted protein, *Campylobacter* invasion antigen B), a mutant that was trafficked into digestive vacuoles and killed at a faster rate relative to the wild type and a +*ciaB* complement strain (*C. jejuni* 11168HΔ*ciaB*+*ciaB*) **[** **Supplementary File 3** (**Supplementary Figure S4** **)]**. A Δ*ciaB* mutant was chosen because (1) it was significantly expressed in our previous RNA-seq analyses (Nasher et al., 2022) and (2) mutation to *ciaB* was shown to seize *C. jejuni*’s ability to secrete other Cia proteins (Konkel et al., 1999).

Mitochondria is a significant source of ROS within cells (Shekhova, 2020). Therefore, we measured ROS levels at different time points to investigate whether *A. castellanii* mitochondria fusion to *C. jejuni* was in response to the infection (**Figure 3E**). We observed a significant (*p* < 0.05) ∼12-fold increase in ROS levels at 1-h post-infection with *C. jejuni*, coinciding with the early stages of *C. jejuni* internalization. Interestingly, ROS was dramatically decreased at 3 h, returning to levels like those in uninfected cells. This pattern persisted at 6 h, suggesting a dynamic regulation of ROS production during infection. We concluded that other mechanisms likely contribute to the close association between the *C. jejuni* and *A. castellanii* mitochondria.

Mitochondria also play a crucial role in regulating cellular calcium levels, a process tightly controlled due to calcium’s role as a catalyst for regulatory cascades impacting various cellular functions (Rossi et al., 2019). Given that we observed significant decrease in transcripts related to calcium homeostasis in our RNA-seq data (**Supplementary Files 1**, **2**) and our observation of the unusual mitochondria phenotype during *C. jejuni* 11168H infection, this prompted us to measure intracellular calcium levels in *A. castellanii* (**Figure 3f**). Significantly (*p* < 0.05) increased calcium levels were observed at 1 h (∼2.21-fold) and 6 h (1.84-fold) post-infection, while at 24 h, we observed a non-significant (∼1.22-fold) elevation in calcium levels.

To investigate whether there is a parallel mitochondrial alteration phenomenon in warm-blooded hosts, we infected human intestinal epithelial T84 cells with *C. jejuni* 11168H and tracked mitochondria after 4 and 24 h of infection (**Figure 3G**). Interestingly, we observed altered host mitochondria dynamics and intimate association with *C. jejuni*, similar to our observation of *A. castellanii* infected with *C. jejuni*. This underscores the profound impact of this pathogen on cellular architecture and also likely their function.

### *C. jejuni* induced *A. castellanii* metabolic rewiring

**Figure 4:**
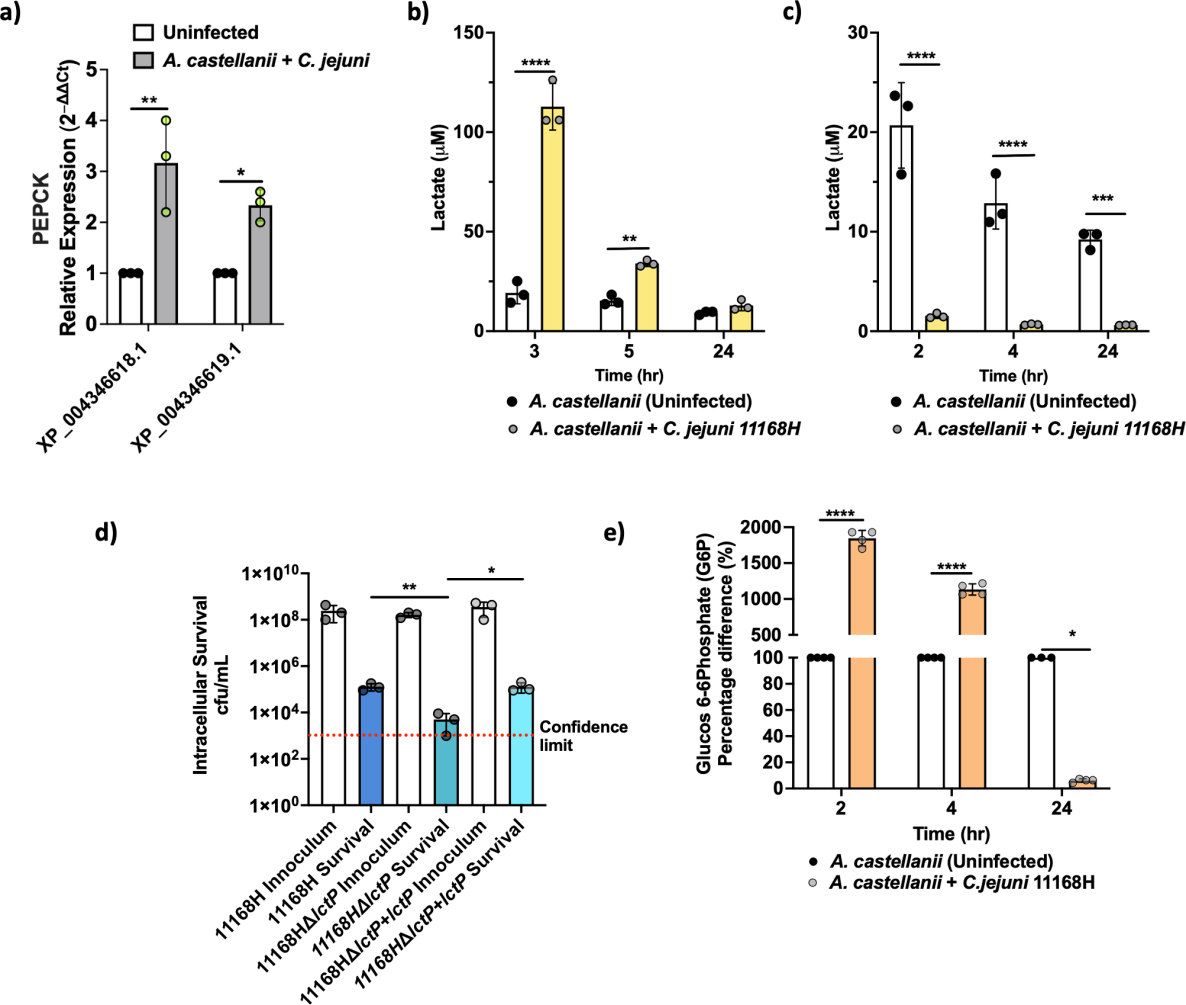
*A. castellanii* switches to Warburg-like metabolism at earlier time points of infection. **(A)** Real-time RT-qPCR of PEPCK encoding genes in *A. castellanii* infected with *C. jejuni* at 4 h post-infection. **(B)** Extracellular lactate concentration 3, 5, and 24 h post-*C. jejuni* infection. *A. castellanii* were infected for 1 h prior to 100 μg/mL gentamycin treatment. **(C)** Extracellular lactate concentration 2, 4, and 24 h post-*C. jejuni* infection (no gentamycin treatment). **(D)** Intracellular survival of *C. jejuni* wild type, Δ*lctP* mutant, and Δ*lctP+lctP* after 4-h infection (including 1-h gentamycin treatment). **(E)** Glucose 6 phosphate (G6P) concentrations in *A. castellanii* infected with *C. jejuni* compared to uninfected. Significance levels indicated as *p < 0.05, **p < 0.001, ***p < 0.0001, ****p < 0.0001. Survival is presented in Log scale. Error bars represent standard deviation (SD). SpectraMax Id5 was used to measure bioluminescence for lactate and G6P was measured by fluorescence (Ex 540/Em 590 nm). *p< 0.05, **p<0.002, *** p<0.0002, ****p<0.0001.

Recent evidence suggests that eukaryotic cells undergoing bacterial infection exhibit distinct metabolic rewiring compared to their uninfected counterparts (Escoll and Buchrieser, 2018). Consistent with this, we noted a significant (*p* < 0.05) upregulation in the expression of pivotal gluconeogenic genes encoding phosphoenolpyruvate carboxykinases (PEPCK), specifically XP_004346619.1 (∼2.46-fold) and XP_004346618.1 (∼2.20-fold), as indicated by our RNA-seq analyses (**Supplementary File 1**). When RT-qPCR was conducted, we observed a 2.33-fold increase for XP_004346619.1 and a 3.16-fold increase for XP_004346618.1 (**Figure 4a**).

To investigate cellular metabolic activity, we monitored gluconeogenesis, the process of generating glucose from non-carbohydrate precursors, particularly during fasting, starvation, or low carbohydrate intake. As an indicator of glycolysis activity, we measured lactate production, a by-product of this metabolic pathway (**Figure 4b**). We observed a notable increase in extracellular lactate in *A. castellanii* infected with *C. jejuni* 2 *h* post-gentamycin treatment, with levels reaching ∼112 μM at 3 h post-infection, significantly higher than the ∼19 μM observed in uninfected cells. This heightened lactate production persisted after 5 h of infection, with infected *A. castellanii* maintaining elevated levels of lactate (∼34 μM), while uninfected cells remained relatively stable at ∼18 μM. Interestingly, lactate levels became comparable between infected and uninfected cells at the 24-h time point.

*C. jejuni* is capable of utilizing lactate from its environment (Stahl et al., 2012); therefore, we measured lactate levels 2, 4, and 24 h post-infection before gentamicin treatment. We observed a significant reduction in lactate levels (**Figure 4c**) in *A. castellanii* with *C. jejuni* compared to uninfected *A. castellanii*. It is possible that under certain circumstances, *A. castellanii* may enhance *C. jejuni* survival outside of the amoeba (Bui et al., 2012).

Previously, we reported a significant (*p* < 0.05) upregulation of *lctP* (a putative lactate permease) in the *C. jejuni* 11168H transcriptome during intracellular survival within *A. castellanii* (Nasher et al., 2022). Here, mutation to *lctP* showed a significant (*p* < 0.05) reduction in *C. jejuni* survival (**Figure 4d**), although this did not lead to a complete failure in intra-amoeba survival, suggesting that other mechanisms of lactate transport into the cell (Thomas et al., 2011; Stahl et al., 2012).

Additionally, glucose-6-phosphate (G6P) levels were assessed; G6P is the first intermediate of glucose metabolism and critical for glycolysis. G6P was substantially increased, reaching up to ∼18.45-fold at 2 h post-infection, and maintaining significant elevation (∼11.33-fold) at 4 h. Intriguingly, at 24 h post-infection, we observed significantly decreased G6P levels (∼16-fold) in *A. castellanii* infected with *C. jejuni* 11168H compared to uninfected cells (**Figure 4e**).

## Discussion

*C. jejuni* is the leading cause of bacterial foodborne gastroenteritis worldwide (Patrick et al., 2018). Investigating the dynamics between *C. jejuni* and Acanthamoeba is important as it illuminates the survival and persistence mechanisms of this pathogen in the environment outside its warm-blooded hosts. In this study, we investigated the response of *A. castellanii* to intracellular infection by *C. jejuni* using RNA-seq.

**Figure 5:**
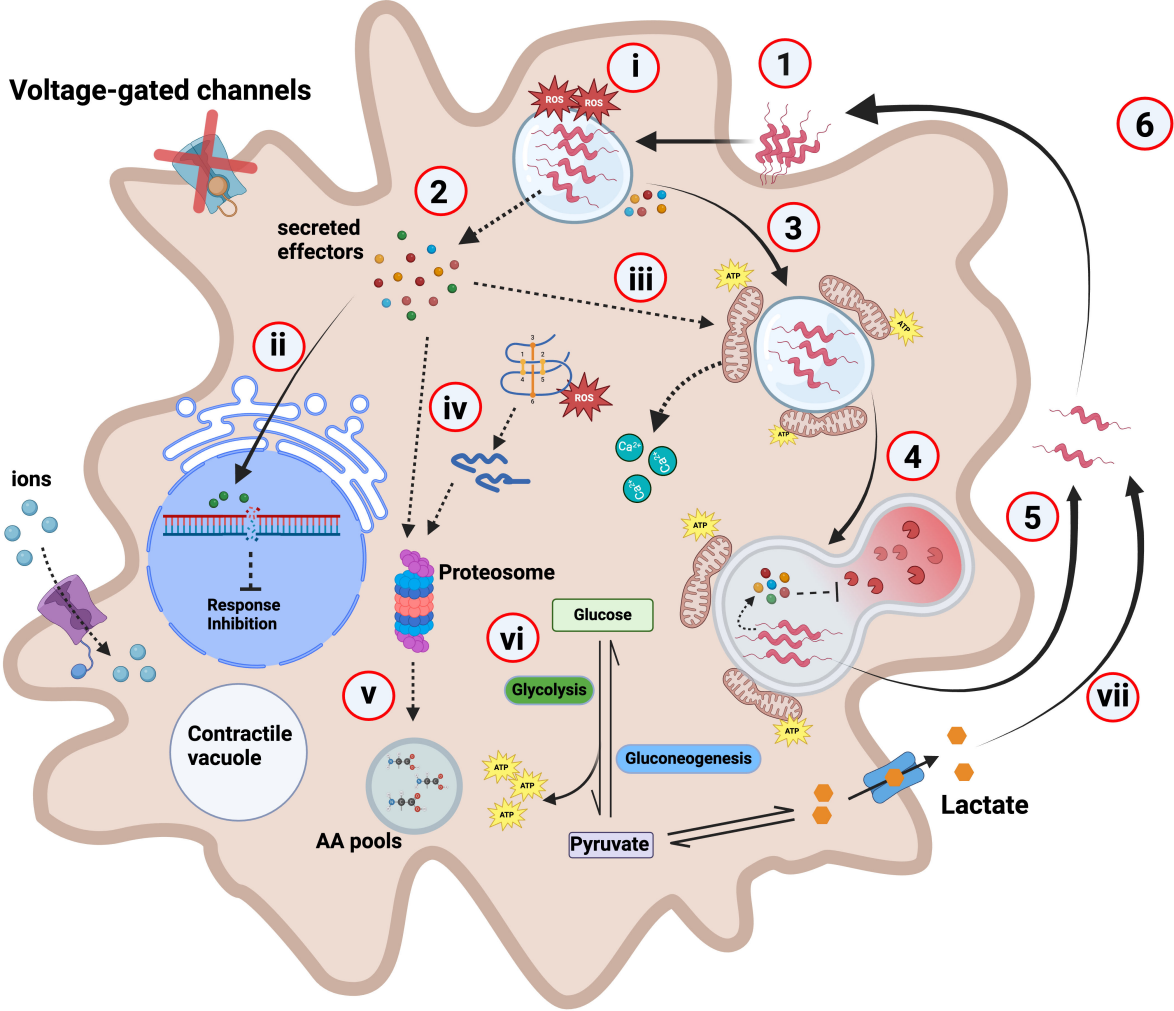
Hypothetical model depicting *C. jejuni* interaction with *A. castellanii*. (1) *C. jejuni* interacts with and enters *A. castellanii* through the glycosylated flagellin (Nasher and Wren, 2023) using an unknown receptor. (2) Upon internalization, *C. jejuni* likely secretes effectors including campylobacter invasion antigens (Cias). (3) These secreted effectors may facilitate *C. jejuni* trafficking and the formation of a *C. jejuni*-containing vacuole (CCV), while interfering with phagosome–lysosome fusion. For example, the effector CiaI has previously been shown to inhibit phagosome–lysosome fusion in mammalian cells (Buelow et al., 2011). Supporting this, our RNA-seq data revealed downregulation of Rab5B—a key early endosomal regulator involved in endosome maturation—suggesting a possible mechanism by which *C. jejuni* impairs endolysosomal trafficking to promote its intracellular persistence. (4) For example, a Δ*ciaB* mutant is digested and cleared at a faster rate relative to the wild type [**Supplementary File 3** (**Supplementary Figure S3**)]. (5) *C. jejuni* are capable of withstanding *A. castellanii* digestion and are exocytosed to the external environment (Nasher et al., 2022). (6) Where these “primed” *C. jejuni* can re-enter the Acanthamoeba transient host at a higher rate (Nasher and Wren, 2022). (i) *A. castellanii* produces high levels of ROS to combat *C. jejuni* infection; this ROS level reduces to similar levels to the uninfected after 3-h infection. (ii) Effectors such as CjeN cause DNA damage, possibly damping immune response and inhibiting other cellular responses. (iii) We hypothesize that a yet-to-be-identified effector possibly induces mitochondrial fusion in *A. castellanii*, leading to its relocation towards the periphery of the vacuole containing *C. jejuni*. Alterations to the mitochondria likely causes calcium influx into the cytosol. (iv) *A. castellanii* upregulates proteasomal complex to degrade *C. jejuni* effectors and damaged endogenous proteins. (v) This generates amino acids (AA) that feed into AA pools. (vi) *C. jejuni* infection rewires *A. castellanii* metabolism, and glycolysis and gluconeogenesis are increased; consequently, this increases ATP production. (vii) Elevated glycolysis results in increased lactate as a by-product, which is then secreted where it is used by extracellular *C. jejuni*. Dashed lines indicate experimentally unproven pathways; red “X” on the voltage-gated channel indicates halted/or reduced activity.

The identified upregulated and downregulated transcripts shed light on the molecular mechanisms that underscore the interactions between *A. castellanii* and *C. jejuni*. Our findings revealed previously unreported interactions and highlight the complex interplay between host and pathogen at the molecular level. This study provides valuable insights into the strategies employed by *C. jejuni* to subvert host cellular processes. Upregulated processes such as DNA replication, cell cycle regulation, and protein folding suggested an active cellular response to infection, likely aimed at repairing DNA damage and maintaining cellular homeostasis. ATPase activity, electron transport chain components, and hydrolase activity indicated an increase in energy production and metabolic activity, possibly to support host defense mechanisms and cellular processes crucial for combating infection. On the other hand, the downregulation of processes related to calcium ion transport, peptidyl-tyrosine phosphorylation, and osmotic stress response may reflect the hijacking of host cellular machinery by *C. jejuni* to create a favorable intracellular environment for its survival. Based on these new data, we provide a hypothetical model of the *C. jejuni* life cycle and its interaction with free-living amoeba (**Figure 5**).

DSBs are particularly harmful lesions to DNA, requiring repair mechanisms to maintain chromosomal integrity (Mehta and Haber, 2014). Errors in this process can lead to mutations, and rearrangements of chromosomes, ultimately contributing to cell death (Cannan and Pederson, 2016). *C. jejuni* endonuclease, CjeN, identified in a previous RNA-seq analysis of intra-amoebic *C. jejuni* (Nasher et al., 2022), has been characterized in this study. Upon *C. jejuni* 11168H infection, CjeN induces DSBs in *A. castellanii*, triggering DNA damage and repair response mechanisms. CjeN degrades both double-stranded DNA and RNA, and its activity was dependent on divalent metal ions Ca^+2^ and Mg^+2^, and Zn^+2^ inhibits its activity. Metal homeostasis is central to host–pathogen interactions (Ammendola and Battistoni, 2022); therefore, it was not surprising to have observed differential regulation of genes associated with several metal ions, including those associated with Ca^+2^, Zn^+2^, and copper (Cu^+2^) homeostasis during *C. jejuni* infection of *A. castellanii*. Although CjeN has a signaling peptide and is likely transported to the extracellular environment, it remains unclear whether it is consistently cleaved and detached from the bacteria or if it remains attached to the surface in some instances, similar to other bacteria (Beiter et al., 2006). However, given that we did not observe close proximity of the bacteria to the nucleus of its amoeba host, we presume that, at least in this instance, Cjen is likely secreted and cleaved. We propose that DSBs caused by CjeN may impair the ability of host cells to mount an effective immune response and interfere with cellular signaling pathways involved in responding to bacterial infections, allowing *C. jejuni* to persist for longer periods. CjeN was previously proposed to be involved in biofilm dispersal (Ramesh, 2020), and it is quite likely that CjeN may also promote *C. jejuni* immune evasion during infection of warm-blooded hosts by degrading neutrophil extracellular traps (NETs) (Rada, 2019).

*C. jejuni* 11168H infection of *A. castellanii* and human T84 epithelial cell line induced alteration to host mitochondria, causing fusion and their relocation to the periphery of *C. jejuni*. Mitochondria play a pivotal role in orchestrating various cellular processes and host defense mechanisms during bacterial infections. Numerous pathogens, *Legionella pneumophila* (Newsome et al., 1985), *Mycobacterium tuberculosis* (Abarca-Rojano et al., 2003), and *Chlamydia* spp (Fischer et al., 2004), manipulate mitochondrial dynamics and functions to enhance their intracellular survival or evade immune detection. Mitochondria dynamic behavior is characterized by continuous fission, fusion, and trafficking. These processes are dictated by the cellular energy demands, wherein mitochondrial fusion is favored in conditions requiring increased ATP production via oxidative phosphorylation (OXPHOS) (Dorn, 2019). Consistent with this, we also observed an increased electron transport chain (ETC) and its activity in *A. castellanii* infected with *C. jejuni*, suggesting increased OXPHOS and ATP production. This may be attributed to *A. castellanii* boosting ATP production to enhance cellular processes involved in combating the pathogen. Conversely, alterations to mitochondria dynamics induced by *C. jejuni* infection could have influenced ETC activity; however, this remains to be elucidated.

Mitochondria are also the main source of cellular ROS (Turrens, 2003). Despite observing a ∼12-fold increase in ROS levels at 1 h post-infection, it was surprising that ROS returned to levels similar to those of uninfected cells by 3 h post-infection. This observation prompted us to reconsider the purpose of mitochondria fusion to *C. jejuni*. While traditionally viewed as a mechanism for antimicrobial activity (Holmbeck and Shadel, 2016), our findings suggest an alternative hypothesis. We propose that the recruitment and fusion of mitochondria to *C. jejuni* may serve a different, yet unknown function, potentially manipulated by the bacteria themselves. This manipulation of mitochondria dynamics and their recruitment can provide *C. jejuni* with advantages such as access to nutrients (Spier et al., 2019). *C. jejuni* exhibits distinctive metabolic characteristics, setting it apart from other enteropathogenic bacteria, notably its limited carbohydrate catabolism. Indeed, the mitochondria serve as a hub for amino acid synthesis, including the production of glutamine, glutamate, proline, and aspartate (Li and Hoppe, 2023). All are capable of supporting *C. jejuni* viability (Stahl et al., 2012) and growth. Previously, we reported significant upregulation of the Peb1 system in intra-amoebae *C. jejuni*, an ABC transporter responsible for the uptake of aspartate and glutamate (Nasher et al., 2022). Mutation in *peb1c* (*cj0922c*), an ATP-binding protein responsible for energy coupling to the peb1ABC transporter, did not exhibit a survival defect **[** **Supplementary File 3** (**Supplementary Figure S5** **)]**, suggesting potential redundancy in the system (Stahl et al., 2012).

In addition to amino acid synthesis and ATP production, mitochondria play a crucial role in regulating cellular calcium homeostasis (Matuz-Mares et al., 2022), which is essential for various cellular functions. Mitochondria act as buffers, sequestering and releasing calcium ions in response to intracellular signals (Matuz-Mares et al., 2022). The increase in cytosolic calcium levels in *A. castellanii* triggered by *C. jejuni*, as demonstrated in previous studies using mammalian cells (Hu et al., 2005), leads to speculation regarding potential connections between calcium homeostasis and the observed alterations in mitochondria during *C. jejuni* infection.

Various studies indicate that bacteria trigger distinct metabolic programs in phagocytes during infection, and this metabolic rewiring is thought to be crucial for bacterial replication and/or survival, as well as host response to the infection (Escoll and Buchrieser, 2018; Price et al., 2020). Here, we present evidence that supports *A. castellanii* rewires its metabolic activities at early stages of the infection. Interestingly, these metabolic activities return to levels comparable to those of uninfected cells as the infection progresses. We noted upregulation of phosphoenolpyruvate carboxykinases (PEPCK), responsible for converting oxaloacetate to phosphoenolpyruvate (PEP), a precursor of glucose (Yang et al., 2009). PEPCK, coupled with enhanced G6P and increased lactate production, suggested a metabolic shift towards glycolysis (Chaudhry and Varacallo, 2018). This metabolic adaptation may be driven by the demand for glucose as an energy source to counter *C. jejuni* infection. These findings are consistent with a metabolic response resembling the “Warburg effect” phenomenon, which is commonly observed in cancer cells and often associated with increased aerobic glycolysis (Escoll and Buchrieser, 2018). Aerobic glycolysis generates less ATP per molecule of glucose compared to OXPHOS but allows for the rapid production of ATP and other essential biomolecules needed to mount an effective response to the infection (Soto-Heredero et al., 2020).

Previously, it was suggested that *A. castellanii* might promote the survival and proliferation of extracellular *C. jejuni* through the mechanism of amoeba-mediated depletion of dissolved oxygen (Bui et al., 2012). Presently, we propose that amoeba lactate production may also play an additional important role in this phenomenon. The increased aerobic glycolysis produced lactate as a by-product, and our data revealed a rapid depletion of extracellular lactate in the presence of *C. jejuni*. This aligns with our previous study, in which we reported a significant upregulation of LctP, an l-lactate permease (Nasher et al., 2022). Therefore, we propose that *C. jejuni* effectively exploits lactate produced by *A. castellanii* as an additional energy source that contributes to its survival within the host and in shared environmental niches (Thomas et al., 2011; Hofreuter, 2014; Sinha et al., 2024). This hypothesis also supports recent findings that showed lactate transport regulation is increased in aerobic environments (Sinha et al., 2024), thereby reinforcing the notion that *C. jejuni* effectively utilizes lactate produced by *A. castellanii* as an additional energy source, contributing to its survival in shared environmental niches. It is also worth noting that some strains of *C. jejuni* are hyper-aerotolerant and can withstand higher levels of oxygen, which may further enhance their ability to survive in oxygen-rich environments (Oh et al., 2015). This suggests that hyper-aerotolerance, in combination with lactate utilization, represents a strategy by which *C. jejuni* can persist in both intra- and extracellular environments, thereby enhancing its overall survival and adaptability in diverse ecological niches.

In conclusion, this study represents a significant advancement in our understanding of the interaction between *C. jejuni* and *A. castellanii* and provides general insight into host–pathogen interactions. It provides evidence that *C. jejuni* manipulates its transient amoeba host to facilitate its survival. The findings enlighten us on the sophisticated strategies employed by *C. jejuni* to persist within host cells and the complex dynamics of microbial pathogenesis. Moreover, this study suggests that the *A. castellanii* model could be utilized to study *C. jejuni* host–pathogen dynamics, offering promising opportunities for further understanding and potentially mitigating the impact of *C. jejuni* infections.

## Methods

### Strains and cultures

*C. jejuni* strain NCTC 11168H (Karlyshev et al., 2002) was stored at −80°C using Protect bacterial preservers. The bacteria were streaked on Columbia blood agar plates with 7% horse blood and grown at 37°C in a microaerobic chamber (85% N2, 10% CO2, 5% O2) for 48 h. They were then grown on CBA plates for an additional 16 h at 37°C before use.

*Acanthamoeba castellanii* acquired from the Culture Collection of Algae and Protozoa (CCAP) 1501/10 (Scottish Marine Institute) were grown to confluence at 25°C in 75-cm^2^ tissue culture flasks containing peptone yeast and glucose (PYG) media (proteose peptone 20 g, glucose 18 g, yeast extract 2 g, sodium citrate dihydrate 1 g, MgSO4 × 7H2O 0.98 g, Na2HPO4 × 7H2O 0.355 g, and KH2PO4 0.34 g in distilled water to make 1,000 mL; pH was adjusted to 6.5). Amoebae were harvested into suspension, and viability was determined by counting using inverted light microscope.

T84 (human intestinal cell line) cells were grown in Dulbecco’s modified Eagle’s medium and Ham’s F-12 (DMEM/F-12) supplemented with 10 % FBS and 1 % non-essential amino acid. The monolayers, ~10^5^, were seeded in 24-well tissue culture plates and were grown up to ~10^6^ in a 5 % CO2 atmosphere and were then infected with *C. jejuni* 11168H at a multiplicity of infection (MOI) of 200 : 1 for 4 h including 1 h of mitochondrial staining (mitochondrial staining is described below in detail).

### *C. jejuni* mutant construction

Isothermal assembly (ISA) cloning was used to generate constructs for *C. jejuni* mutagenesis. Genes of interest were disrupted by inserting antibiotic resistance cassette [encoding kanamycin resistance (Kan^r^)] or Apramycin (Ap^r^) within the target reading frame. Constructs containing ~500-bp upstream and downstream flanking regions of the target genes were assembled using the HiFi DNA Assembly Cloning Kit (NEB) and cloned into *Escherichia coli* DH5α via heat shock transformation, following the manufacturer’s protocol. Transformants were selected on LB agar containing apramycin (50 µg/mL) and ampicillin (100 µg/mL). The plasmid backbone used for cloning was pGEM^®^-3Zf(–) (Promega). Plasmids were purified from *E. coli* and introduced into *C. jejuni* 11168H by electroporation at room temperature. Following electroporation, cells were recovered on CBA plates and incubated at 37°C under microaerophilic conditions for 5 days to allow chromosomal integration via double homologous crossover. Recombinant mutants Δ*cdtABC* (Ap^r^) and ΔlctP (Kan^r^) were selected using appropriate antibiotics and verified by PCR. Primer sequences are listed in **Supplementary File 3** (**Supplementary Table S3**).

Complementation of Δ*cjeN*, Δ*lctp*, and Δ*ciaB* was generated using the plasmid pRRA (A = Apramycin) as previously described (Karlyshev and Wren, 2005; Nasher and Wren, 2023). Briefly, the wild-type *cjeN*, *lctP*, and *ciaB* genes with an intact native promoter were amplified and the fragments were inserted into plasmid pRRA (Ap^r^) and electroporated into *C. jejuni*.

### *C. jejuni* recombinant protein expression and purification

Recombinant CjeN_his6_ (Cj0979c) and its variant CjeN_Thr69Ser77Thr111his6_ were cloned into pET21a^(+)^ plasmid using a NEBuilder HiFi DNA assembly cloning kit. The proteins were overexpressed in *E. coli* strain BL21(DE3) grown in LB broth containing 150 μg/mL ampicillin at 37°C overnight. Cells were harvested, resuspended in lysis buffer with protease inhibitors, and lysed. The supernatant was incubated with Ni-NTA agarose, and the recombinant protein was eluted with lysis buffer containing 400 mM imidazole. Primers used for cloning are listed in **Supplementary File 3** (**Supplementary Table S3**).

### RNA extraction and differential gene expression analysis

*A. castellanii* was infected with *C. jejuni* 11168H for 4 h, including 1 h of gentamicin treatment to kill extracellular bacteria. Infected and uninfected cells were centrifuged at 4000 rpm for 5 min to pellet the cells. The supernatant was discarded, and the cells were washed three times with PBS. Total RNA was then extracted using TRIzol (Invitrogen), followed by RNA quantification and quality assessment using a Bioanalyzer (Agilent). rRNA depletion, library preparation, and sequencing with NovaSeq were carried out by GENEWIZ (Azenta Life Sciences). *A. castellanii* infected with *C. jejuni* strain 11168H was defined as the “test group” and uninfected *A. castellanii* was defined as the control (incubated in the absence of bacteria) after a total of 4 h. Illumina 150-bp paired-end reads were trimmed using Trimmomatic V 0.39; with parameters leading: 3, trailing: 3, and MINLEN: 36, TruSeq3-PE adapter removal was also conducted. Abundance estimates were quantified using Kallisto (v0.46.1) with default parameters against reference sequence, Acastellanii.strNEFF v1 GCA_000313135.1. Differential gene expression analysis was conducted using edgeR (empirical analysis of DGE in R) in R with an adjusted *p*-value cutoff of <0.05, and a log fold change cutoff of >2.

For GO annotation and enrichment analysis, Illumina RNA-seq reads derived from infected and uninfected *A. castellanii* were first subjected to *De Novo* assembly using a Trinity v2.15.1 assembler. The *A. castellanii* Neff strain genome was used as the reference. The resulting transcripts were quantified using RSEM (RNA-seq by Expectation-Maximization) (Langmead and Salzberg, 2012) followed by differential expression analysis using edgeR on OmicsBox (Biobam). GO mapping was done using OmicsBox (Biobam).

### Gene expression by real-time RT-qPCR

To validate our RNA-seq experiments, total RNA for RT-qPCR was extracted from independent biological replicates that were subjected to identical experimental conditions as those used for RNA-seq experiments. cDNA synthesis and genomic DNA elimination were performed using the RT2 Easy Frist Strand kit (Qiagen). Quantification of gene expression was achieved by real-time RT-qPCR using Sybr™ Green real-time PCR master mix using primers generated using Primer Quest (IDT) **[** **Supplementary File S1** (**Supplementary Table S4** **)]**. Relative gene expression for the different genes was calculated from the crossing threshold (Ct) value according to the manufacturer’s protocol (2^−ΔΔCt^) after normalization using the 18s endogenous control (Nachamkin et al., 1993).

### Survival assay

Invasion and survival assays were performed as previously described (Nasher and Wren, 2022). Briefly, *C. jejuni* 11168H and its mutants were incubated with a monolayer of approximately 10^6^ A*. castellanii* at a MOI of 100:1 for 3 h at 25°C. The monolayer was washed 3× with phosphate buffer saline solution (PBS) and incubated for 1 h in PYG media containing 100 μg/mL of gentamicin.

*C. jejuni* cells were harvested by lysing amoebae with Triton X-100, followed by centrifugation. The bacterial pellet was resuspended in PBS and colony-forming units were enumerated on CBA plates after incubation at 37°C for up to 72 h in a microaerobic environment.

### *Galleria mellonella* infection model

*C. jejuni* strains 11168H wild type, 11168HΔ*cjeN*, or 11168HΔ*cjeN*+*cjeN* were suspended in PBS to an OD_600_ of 0.1. A 10-µL volume (~10^6^ bacteria) was injected into the larvae’s right foremost leg and incubated at 37°C. Ten larvae of similar weight were used per strain in each of three biological replicates. Mortality was recorded at 24-h intervals for 72 h.

### DNA double-stranded breakages and mitochondria imaging

*A. castellanii* cells were infected with *C. jejuni* 11168H wild type and its mutants, then fixed with 4% paraformaldehyde and permeabilized with 0.1% Triton X-100 in PBS. Samples were mounted with Fluoroshield™ containing DAPI and imaged using an inverted confocal microscope. For assessing DNA breakages, *A. castellanii* were infected with *C. jejuni* strains or CjeN_his6_-coupled latex beads for 4 h. *A. castellanii* were stained with anti-γH2AX antibody followed by Alexa Fluor^®^ 488 secondary staining. All CjeN *in vitro* nuclease activity assays were performed in Tris-HCl (pH 7.5) buffer containing 130 µM calcium (Ca^+2^), 5 mM magnesium (Mg^+2^), and 5% glycerol at 37°C for 30 min and samples were ran on 1% Agarose gel.

Mitochondria imaging was performed at specified intervals following infection. *A. castellanii* were incubated with Mitobrilliant™ 646 for 1 h. Cells were washed and fixed before permeabilization and imaging.

### Imaging and microscopy

Confocal laser scanning microscopy and super-resolution Airyscan images were obtained using an inverted Zeiss LSM 880 confocal microscope (Zeiss, Berlin, Germany). Images were taken at 5-s intervals for time lapse experiments. Alexa Fluor^®^ 488 was monitored in the green region [excitation (Ex) and emission (Em) wavelengths are 488/510 nm]. Mitobrilliant™ 646 was monitored in the red region (Ex/Em = 655/668 nm). 4′,6-diamidino-2-phenylindole (DAPI) was monitored in the blue region (Ex/Em = 355/405).

### Calcium and reactive oxygen species quantification assay

Calcium dysregulation was monitored using the Fluo-4 NW Calcium Assay Kit (Thermo Fisher Scientific, USA) according to manufacturer’s instructions. *A. castellanii* were infected with *C. jejuni* 11168H in a 96-well plate. Cells were washed 3× with PBS, and 100 µL of the dye loading solution was added to each sample well. The plate was incubated at 25°C for 1 h prior to reading using SpectraMax iD5 (Molecular Devices). Fluo NW was measured at Ex 495/515 nm.

ROS was quantified using CellROX™ Deep Red (Thermo Fisher) following the manufacturer’s instructions. Briefly, after infection, *A. castellanii* cells were washed and 5 μM of CellROX Red reagent was added to the cells for 30 min. Cells were washed three times with PBS before analyzing with SpectraMax iD5. CellROX™ Deep Red fluorescent was measured at Ex 644/Em 655 nm.

### Lactate and glucose-6-phosphate assay

To determine lactate and G6P concentrations in *A. castellanii* infected with *C. jejuni*, cells were infected as described above for 2, 4, and 24 h. Following infection, cells were homogenized and subjected to deproteinization. The samples were analyzed for lactate using the Lactate-Glo™ Assay Kit (Promega), and the Amplite^®^ Fluorimetric Glucose-6-Phosphate Assay Kit (AAT Bioquest) was used to measure G6P concentrations according to the manufacturer’s instructions with a SpectraMax iD5. Lactate standards provided in the kits were used to determine absolute concentrations.

### Statistical analysis

All experiments presented are at least three independent biological replicates. RNA-seq analysis was performed in R using the combined data generated from the bioinformatics. The evaluation of *A. castellanii* interaction with *C. jejuni* was based on at least 200 amoeba cells per experiment. Differentially expressed genes were considered significant when the *p*-value of three independent biological experiments was below <0.05. All other data were analyzed using Prism statistical software (Version 9, GraphPad Software, San Diego, CA, USA), and statistical significance was considered when the *p*-value was <0.05. All values are presented as standard deviation.

## Data Availability

The data that supports the RNA-Seq findings of this study are available in the Gene Expression Omnibus (GEO) under data set identifier GSE262802. Link: https://www.ncbi.nlm.nih.gov/geo/query/acc.cgi?acc=GSE262802.

## Supplementary material

Supplementary File 1 All differential regulated genes in *A. castellanii* infected with *C. jejuni*.

Supplementary File 2 All GO Term enrichment genes in *A. castellanii* infected with *C. jejuni*.
