# Supplementary material for: Acanthamoeba castellanii as a model for unveiling Campylobacter jejuni host–pathogen dynamics
Source: Front Cell Infect Microbiol. 2025 Jun 18;15:1583830. doi: 10.3389/fcimb.2025.1583830 (PMC12213738; doi:10.3389/fcimb.2025.1583830)
Supplement: Supplementary file 3 [file SupplementaryFile3.pdf]

Supplementary File 3

Acanthamoeba castellanii as a model for unveiling *Campylobacter jejuni* host-pathogen dynamics.

Fauzy Nasher[1*], Burhan Lehri[1], Richard Stabler[1], Brendan W. Wren[1*].

[1]Department of Infection Biology
London School of Hygiene and Tropical Medicine
Keppel St, London WC1E 7HT

***Correspondence:** Fauzy Nasher (fauzy.nasher1@lshtm.ac.uk) and Brendan W. Wren (brendan.wren@lshtm.ac.uk)

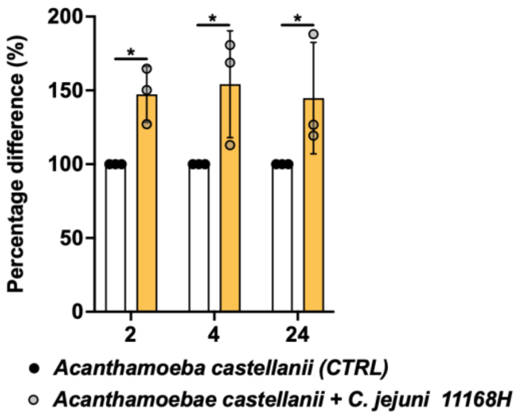

Figure S1. Proteosome 20s activity assay. Proteosome (20S) activity was monitored using Amplite® Fluorimetric Proteasome 20S Activity Assay Kit Green Fluorescence (AAT Bioquest) according to manufacturer's instructions. Amplite® Fluorometric 20S Proteasome Assay Kit uses LLVY-R110 as a fluorogenic indicator for proteasome activity. Briefly, amoeba cells were infected with *C. jejuni* 11168H in a 96 well plate as described above, cells were washed 3x with PBS and 100 µL proteasome working solution was added per well. The plate was incubated at 25°C for 2 h and fluorescence intensity was monitored at Ex/Em = 490/525 nm using SpectraMax iD5. Significance levels are indicated as follows: *$p<0.05$; error bars represent standard deviation (SD).

Multiple Sequence Alignment

```
CjeN      ---------MRINYKKIFNLRKLLSD------PKK--LFSV--LIFTLVVVFIQ-NY---      37
Nuc1      MTEYLLSAGICMAIVSILLIGMAISNVSKGQYAKRFFFFATSCLVLTL--VVVSSLSSSA      58
YncB      --------------------------------MKK-ILISMIAIVLSITLAACGSNHAAK      27
                                           *:  :::   :::::  .

CjeN      --------------------------------IAQNSSFEGKVVRIIDGDTIEVNHENK      64
Nuc1      NASQTDNGVN-RSGSEHPTVYSATS--------TKKLHKEPATLIKAIDGDTVKLMYKGQ      109
YncB      NHSD-SNGTEQVSQDTHSNEYNQTEQKAGTPHSKNQKKLVNVTLDRAIDGDTIKVIYNGK      86
                                             :      .: : *****::: ::.:

CjeN      LARIRFFGIDAPELKQSF------GKQSKEALSRIL-SGKQVEIIY---KNKDTYGRIVA      114
Nuc1      PMTFRLLLVDTPETKHPKKGVEKYGPEASAFTKKMVENAKKIEVEFDKGQRTDKYGRGLA      169
YncB      KDTVRYLLVDTPETKKPNSCVQPYGEDASKRNKELV-NSGKLQLEFDKGDRRDKYGRLLA      145
             .* : :*:** *:        * ::.   ..:: .. :::: :   .. *.*** :*

CjeN      IVKLNDVDINRFLVSKGYAWADTY------YSNAYTKEQENAKKNHLGLWKESNPIEPYK      168
Nuc1      YIYADGKMVNEALVRQGLAKVAYVYKPNNTHEQLLRKSEAQAKKEKLNIWSEDNADSGQ-      228
YncB      YVYVDGKSVQETLLKEGLARVAYVYEPNTKYIDQFRLDEQEAKSDKLSIWSKSGYVTNRG      205
           :  :.  ::. *: :* * .         : :    .: :**.::*.:*.:..

CjeN      WRKHNKF  175
Nuc1      -------  228
YncB      FNGCVK-  211
```

Figure S2. Multiple Sequence Alignment of *C. jejuni* CjeN, *S. aureus* Nuc1 and *B. subtilis* YncB protein sequences. Green indicates conserved active sites Arg 69 and Glu77 and Argi 111 while blue indicate metal binding sites, Asp 55, 74 and Ala 75. Clustal omega was used for alignment.

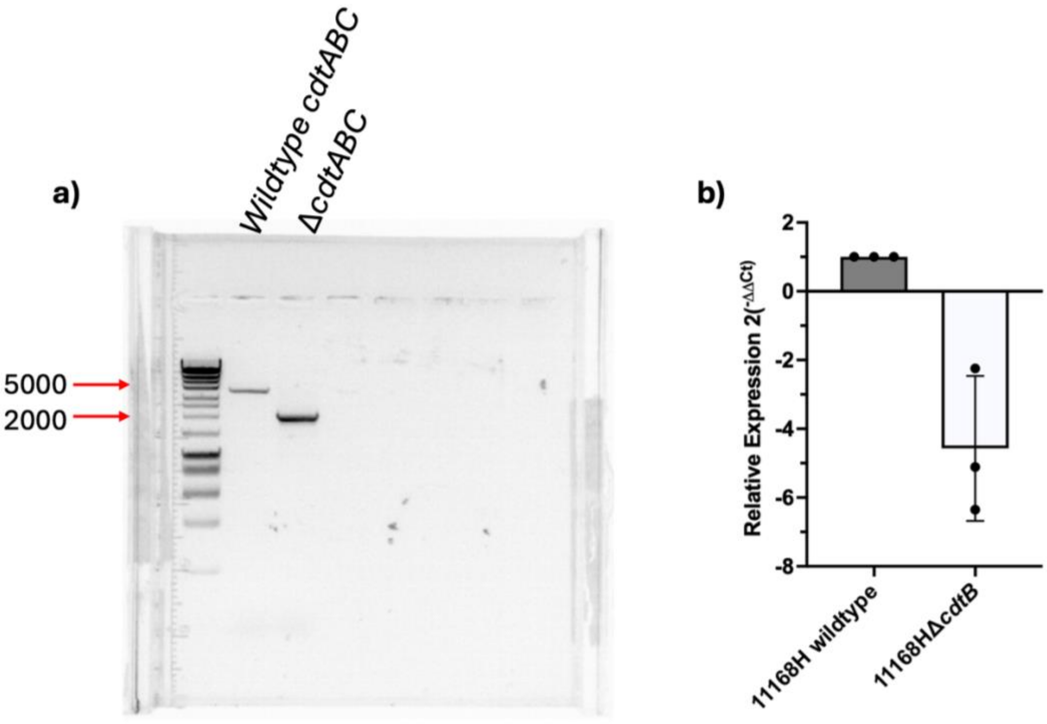

Figure S3: *C. jejuni* 11168HΔ*cdtABC* mutant. a) Agarose gel (1%) electrophoresis showing PCR products of 11168H wildtype and its Δ*cdtABC* mutant (HyperLadder^{TM} 1kb); **b)** RT-qPCR showing expression levels of Δ*cdtABC* mutant relative to the wildtype after normalization with *gyrA* endogenous control.

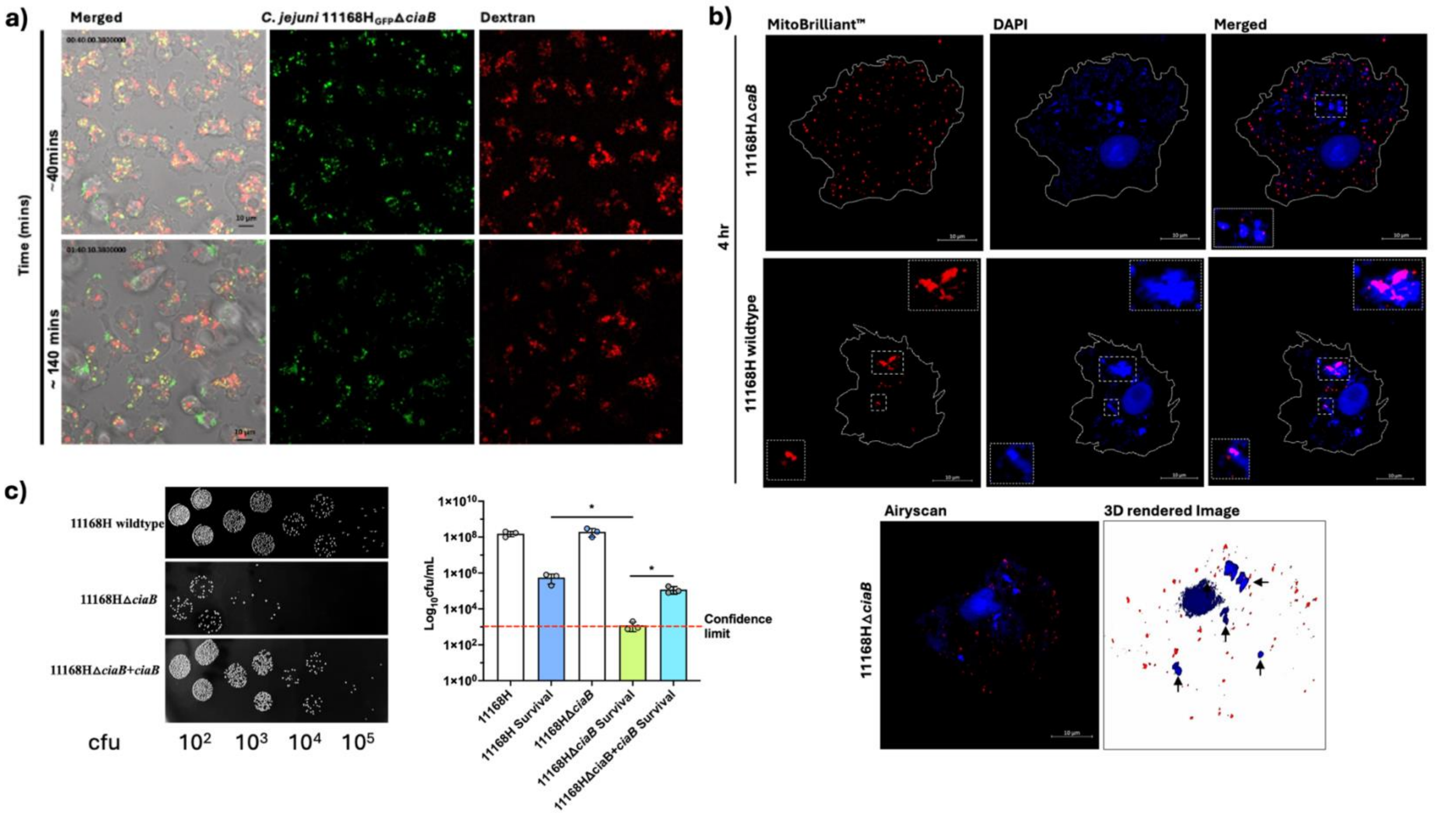

Figure S4: CiaB mutant is trafficked to digestive vacuoles at a faster rate than the wildtype and ciaB mutant is not associated with host mitochondria. a) Time lapse imaging showing *C. jejuni* 11168HΔ*ciaB* mutant ~40 mins and 1 hr 40 mins post infections; interestingly ciaB mutants are trafficked to digestive vacuole at a faster rate than what we usually observe [1]. **b)** CiaB mutant was not associated with host mitochondria and **c)** colony forming units (cfu) of *C. jejuni* 11168H wildtype relative to its Δ*ciaB* and Δ*ciaB*+*ciaB* mutant after 4 hr infection (including 1 hr gentamycin treatment), wildtype strain showed 2-fold survival higher than the Δ*ciaB* mutant strain. Mutants were constructed as described in the methods section. Arrows indicate bacteria. Error bars are presented at standard deviation (SD); *p<0.05.

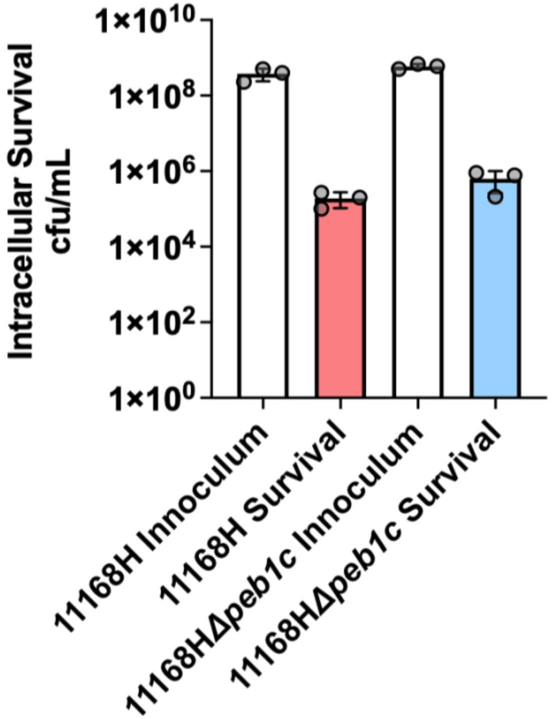

Figure S5: *peb1c* mutant intracellular survival was similar to that of the wildtype strain. *C. jejuni* 11168H Δ*pe1bC* mutant construct was obtained from was acquired from obtained from the Campylobacter Resource Facility (http://crf.lshtm.ac.uk/wren_mutants.htm, accessed on 14 April 2024) and strain 11168H was naturally transformed using the previously described biphasic method [2]. Survival assay was performed as described in the methods.

Table S3: Primers used to make mutants and recombinant proteins.

Primer Name	Sequence	
	**Mutagenesis Primers**	
cdtABC_F1	**Fwd:** 5-ccggggatcctctagagtcggcggaaaattataatgaaattta-3	**Rvs:** 5- gcccaaaccgttaaagctgc -3
cdtABC_Apr	**Fwd:** 5- cggtttgggcgtaacaaggtaaccgtag-3	**Rvs:** 5-aaggtttttattactttgtactctagggc-3
cdtABC_F2	**Fwd:** 5-tacaaagtaatcaggattagaacatttatcc-3	**Rvs:** 5-agcttgcatgcctgcaggtctgcaaggggctattccaaagc-3
CiaB1_F1	**Fwd:** 5-cccggggatcctctagagtctaaataaatgctgataagcttaaag-3	Rvs: 5-ccttgttacgaaactcatataacgcattatttc-3
CiaB_Kanr	**Fwd:** 5-Atatgagtttcgtaacaaggtaaccgtag-3	**Rvs:** 5-tttcacaaaattactttgtactctagggc-3
CiaB2_F2	**Fwd:** 5-tacaaagtaattttgtgaaattgaagataatatttttc-3	**Rvs:** 5-agcttgcatgcctgcaggtctatttcctataagctcacttac-3
lctP_F1	**Fwd:** 5-cccggggatcctctagagtcgttatgtgcaatttacaaat-3	**Rvs:** 5-taaaagtgcagcggttatag-3
lctP_Kanr	**Fwd:** 5-ctataaccgctgcacttttataaccgtag-3	**Rvs:** 5-aaagcatcacctgtgtgtgcttactttgtactctagggc-3
lctP_F2	**Fwd:** 5-gcacacacag gtgatgcttt-3	**Rvs:** 5-agcttgcatgcctgcaggtctaggtacacctttatcttca-3
Δ*lctP*+*lctP* (comp)	**Fwd:** 5-acaccaattgaactaatgattataaccc-3	**Rvs:** 5-acactctagattgttgaaataaaacttaaa-3
Δ*ciaB*+*ciaB*	**Fwd:** 5-acaccaattggttctcccatatctctcatt-3	**Rvs:** 5-acactctagaagtcataaaagctcctttgt-3
Δ*cjeN*+*cjeN*	**Fwd:** 5-acaccaattggtttaaaattttctataacat-3	**Rvs:** 5-acactctagaaatagcaccattaaagtata-3
	**Recombinant protein Primers**	
*CjeNc$_{his6}$	**Fwd:** 5-ctttaagaaggagatatacatatgCAAAATTCTAGTTTTGAAGGAAAAG-3<br>**RVS:** 5-agtggtggtggtggtggtgctcgagGAATTTATTGTGTTTTCTCCATTTATAAG-3	
*CjeN$_{Thr69Ser77Thr111his6}$	**Fwd1:** 5-ctttaagaaggagatatacatatgCAAAATTCTAGTTTTGAAGGAAAAG-3<br>**Rvs 1:** 5- tggtgcatctATACCGAAAAAGCTTATTCTAG -3<br>**Fwd 2:** 5-ttttcggtatAGATGCACCACAACTTAAAC-3<br>**Rvs2:** 5-tttacaatagCAACAATGCTACCATAAGTATC-3<br>**Fwd 3:** 5-agcattgttgCTATTGTAAAGCTTAATGATGTTG-3<br>**Rvs3:** 5-agtggtggtggtggtggtgctcgagGAATTTATTGTGTTTTCTCCATTTATAAG-3	

Fwd: forward; Rvs: reverse; F1: fragment 1; F2: fragment 2; Comp: Complementation; *Lower case indicate overlap.

Table S4: RT-qpCR primers

Primer Name	Sequence
XP_004336746.1	Fwd: 5-GCTTGCGTTGGGTCAGT-3<br>Rvs: 5-CAGCTTGATGTCGTCCTTGT-3
XP_004349665.1	Fwd: 5-CTACTCCTCAATGTGCTCCAAC-3<br>Rvs: 5-GCAGTCGGCCATAGTCATTT-3
XP_004334023.1	Fwd: 5-GAGGAGGAGTTTATGACG-3<br>Rvs: 5-GAGGGCAATTCCAATCAG-3
XP_004336998.1	Fwd: 5-AACCAAAGCGAGTCGTACC-3<br>Rvs: 5-ATCTCCACTCCCTCCTCTTC-3
XP_004352909.1	Fwd: 5-TACCTCCTCTCCTCGTTCAA-3<br>Rvs: 5-GAATCGTGTTCGGTTCTCAAAG-3
XP_004346566.1	Fwd: 5-ACAAGCCCAAGACTCTGATG-3<br>Rvs:5-TCTGCACCTGGTAGATGGTA-3
XP_004346021.1	Fwd: 5-CTTTAAGCAGCTCCTCCAGTC-3<br>Rvs: 5-CCCTCGGTCATGAAGATGTTT-3
XP_004337009.1	Fwd: 5-AGCGCAATGACAGGTGAA-3<br>Rvs: 5-AATGCACACCCGGCTAAA-3
XP_004342027.1	Fwd: 5-TCCGCGAGATCAGGAAGTA-3<br>Rvs: 5-CTGGAACCTCAGATCAGTCTTG-3
XP_004346618.1	Fwd: 5- CCACAACATCATTCGCAACC-3<br>Rvs: 5- GTGCTTGCCGGACTTACA-3
cdtB	Fwd: 5-CGCGTTGATGTAGGAGCTAAT-3<br>Rvs: 5-GTCTTGAAACTGTAGTAGGTGGAG-3
gyrA	Fwd: 5-AGTAATACGTGGCACATCAAATTTACTTCTAAT-3<br>Rvs: 5- GCAGAATTAATGAAAGAAATTGCAAGACTTG-3

Fwd: forward
Rvs: reverse

Reference

1. Nasher, F. *et al.* (2022) Survival of Campylobacter jejuni 11168H in Acanthamoebae castellanii Provides Mechanistic Insight into Host Pathogen Interactions. *Microorganisms* 10. 10.3390/microorganisms10101894
2. Wang, Y. and Taylor, D. (1990) Natural transformation in Campylobacter species. *Journal of bacteriology* 172, 949-955
